# Interactive Regimes of Reduced Irrigation and Salt Stress Depressed Tomato Water Use Efficiency at Leaf and Plant Scales by Affecting Leaf Physiology and Stem Sap Flow

**DOI:** 10.3389/fpls.2019.00160

**Published:** 2019-02-28

**Authors:** Hui Yang, Manoj K. Shukla, Xiaomin Mao, Shaozhong Kang, Taisheng Du

**Affiliations:** ^1^Center for Agricultural Water Research in China, China Agricultural University, Beijing, China; ^2^Plant and Environmental Sciences Department, New Mexico State University, Las Cruces, NM, United States

**Keywords:** reduced irrigation, salt stress, tomato, water use efficiency, sap flow, soil moisture

## Abstract

Interactive effects of reduced irrigation and salt stress on leaf physiological parameters, biomass accumulation, and water use efficiency (WUE) of tomato plants at leaf and whole plant scales were investigated in a field experiment during 2016 and a greenhouse experiment during 2017. Experiment utilized two irrigation regimes (full, 2/3 of full irrigation) and four soil salt regimes (0, 0.3, 0.6, 0.9% in 2016 season; and 0, 0.2, 0.3, 0.4% in 2017 season). Three salts, sodium chloride, magnesium sulfate, and calcium sulfate (mass ratio of 2:2:1), were homogeneously mixed with soil prior to packing into containers (0.024 m^3^). Li-COR 6400 was used to measure tomato leaf physiological parameters. Instantaneous water use efficiency (WUE_ins_, μmol mmol^−1^) and intrinsic water use efficiency (WUE_int_, μmol mol^−1^) were determined at leaf scale, yield water use efficiency (WUE_Y_, g L^−1^), and dry biomass water use efficiency (WUE_DM_, g L^−1^) were determined at whole plant scale. Plants irrigated with 2/3 of full irrigation with zero soil-salt treatment had higher dry biomass and yield per plant, resulting in the highest WUE_DM_ and WUE_Y_ at whole plant scale. Increasing soil salinity decreased dry biomass and yield, leading to greater decreases in whole plant WUE_DM_ and WUE_Y_ under both irrigation treatments. At full irrigation, no decreases in stomatal conductance (g_s_, mol m^−2^ s^−1^) and slight increase in photosynthetic rate (P_n_, μmol m^−2^ s^−1^) led to higher WUE_int_ at leaf scale during both years. Under full and reduced irrigation, increasing soil salt content decreased P_n_ and transpiration rate (T_r_, mmol m^−2^ s^−1^) and led to reductions in WUE_ins_ at the leaf scale. However, compared to full irrigation, reduced irrigation improved WUE_ins_ with a significant decline in T_r_ in no salt and 0.3% soil-salt treatments during both years. For soil salt content of 0.6%, stomatal limitation due to salt stress resulted in higher WUE_int_, but soil salt content of 0.9% decreased WUE_int_ due to non-stomatal limitation. Soil salt content significantly decreased sap flow, with the maximum variation of daily sap flow per plant of 7.96–31.37 g/h in 2016 and 12.52–36.02 g h^−1^ in 2017. Sap flow rate was linearly related to air temperature (T_a_, °C), solar radiation (R_s_, W m^−2^), and vapor pressure deficit (VPD, kPa). These results advance knowledge on tomato response to abiotic stresses and could improve management of tomato production in water- and salt-stressed areas.

## Introduction

Appropriate water-saving irrigation regimes are needed to alleviate the threat of water shortage and severe drought on food security under increasing population worldwide (Wei et al., 2016), especially in ecologically fragile arid and semi-arid areas. More than 6% of the world's land is subject to salinity problems (Unesco Water Portal, 2007), and the use of water-saving strategies could exacerbate secondary salinization. Approximately 20% of irrigated land was affected by salinity where crop yields were notably reduced (Qadir et al., 2014). Therefore, soil salinity measurements must precede implementation of water-saving irrigation regimes (Reina-Sánchez et al., 2005). Increases in the duration of droughts have necessitated the use of lower quality groundwater to supplement irrigation in semi-arid regions (Flores et al., 2016, 2017; Baath et al., 2017). Understanding plant responses to coupled abiotic stresses of water and salinity, and the underlying mechanisms of improving WUE from leaf to whole plant scale, would be useful to stabilize crop performance and production under drought and saline conditions in a changing climate.

Salinity-induced morphological and physiological changes in plants are mostly identical to drought during osmotic stress phase (Munns, 2002). Drought and salinity stresses cause progressive reductions of water use, leaf growth, and yield via restriction on stomatal apertures to mediate leaf photochemistry and carbon metabolism (Negrão et al., 2017). Under salinity stress, plants could suffer due to salt-specific effects of ion toxicity (Deb et al., 2013; Farooq et al., 2015). Most of the experiments reported so far were conducted under simulated conditions of using either hydroponic culture with different gradients of nutritive solutions (Albaladejo et al., 2017) or soil irrigated with different levels of saline solutions of NaCl (Ahmed et al., 2013; Galli et al., 2016), NaCl and CaCl_2_ (Katerji et al., 2011), or brackish groundwater (Flores et al., 2016; Baath et al., 2017).

It is more realistic for plant roots to be exposed to multiple salts simultaneously due to the specific ion effects as wells as competitions among ions (Farooq et al., 2015), but very few studies have been conducted in soil containing salt. Schiattone et al. (2017) mixed two salts (NaCl and CaCl_2_) with soil to investigate water use and rocket crop performance under different salt-stressed conditions. The results showed that the increasing soil salt content decreased leaf size and numbers, water use, and yield. Faster uptake and transport from roots to the shoots of ions in solution caused symptoms to occur early in tomato leaves (Albaladejo et al., 2017). Na^+^ interfered with K^+^ uptake causing disturbance in stomatal regulations (Siddiqi et al., 2011) and also stimulated sulfate uptake of safflower plant (Patil, 2012).

Plant acclimation to water stress is the result of osmotic adjustment by chemical growth regulators in roots which maintain plants water status with little influence on photosynthetic rate (Martínez et al., 2007; Chaves et al., 2009; Du et al., 2015; Negrão et al., 2017). In addition, plant adaptation to salinity causes adjustments in ion uptake, extrusion, and sequestration as well as synthesis of compatible solutes to maintain cellular homeostasis (Chaves et al., 2009). Some salt-tolerant species are better able to maintain a longer greenness and photosynthetic process under high levels of Na^+^ concentration in tissues (Flores et al., 2016; Negrão et al., 2017). However, response to individual stress factors cannot be isolated from plant response (Mittler, 2006).

Another challenge is linking changes in leaf physiology to WUE at leaf and whole plant scales. Reduced irrigation has been reported to improve WUE at plant scale by maintaining yield (Chen et al., 2013; Cosić et al., 2015; Yang et al., 2017). WUE at leaf scale is impacted by external factors, e.g., VPD and soil water content, and internal factors, e.g., stomatal conductance, leaf mesophyll conductance, and leaf water deficit (Chaves et al., 2009; Niu et al., 2011). Instantaneous water use efficiency (WUE_ins_, μmol mmol^−1^) is reported to decrease with increasing rainfall (Farquhar and Sharkey, 1982) while intrinsic water use efficiency (WUE_int_, μmol mol^−1^) is improved by sustaining P_n_ or decreasing g_s_ (Wang et al., 2010).

Tomato is moderately tolerant to salinity, with a threshold saturated paste EC of 1.3~6 dS m^−1^ (Maggio et al., 2004). The marketable yield and dry matter of tomato decrease with salinity (Reina-Sánchez et al., 2005). Dry biomass water use efficiency (WUE_DM_, g L^−1^) of tomato plants did not differ at 35 and 70 mM NaCl compared to control (Romero-Aranda et al., 2001). In contrast, Reina-Sánchez et al. (2005) found that WUE_DM_ of four tomato cultivars slightly improved, while yield water use efficiency (WUE_Y_, g L^−1^) decreased with increasing salinity (Zhang et al., 2016). However, water use efficiency (WUE_ins_ and WUE_int_) at leaf scale for tomato species under salt-stressed conditions are still unknown.

Generally, sap flow rate is affected by various internal and external factors. Internal factors refer to plant water status, i.e., canopy structure, stomatal aperture, stem hydraulic structure, and hydraulic conductivity of roots; and external factors including solar radiation (R_s_, W m^−2^) and VPD (De Swaef and Steppe, 2010; Liu et al., 2010). Only a few studies have documented that sap flow of tomato plants significantly decreased in deficit-irrigated treatments (Liu et al., 2010; Qiu et al., 2015; Mao et al., 2017).

Tomato is widely planted in northwest China. However, with increasing tomato consumption and decreasing water resources in northwest China, greenhouse tomato cultivation has shown a large potential. Therefore, in this study, one field experiment and one greenhouse experiment were conducted in 2016 and 2017, respectively. Our hypotheses were that reduced irrigation coupled with salt stress will decrease tomato sap flow rate and the WUE will improve under water stress with/without mild salt stress at leaf and plant levels. The objectives were to (1) investigate the influence of simultaneous water and salt stresses on WUE_ins_ and WUE_int_ at leaf and plant scale, (2) evaluate actual transpiration of tomato under different water and salt treatments, and (3) determine tomato WUE from leaf scale to plant scale under different water and salt stresses.

## Materials and Methods

### Experimental Setup

Two experiments were carried out, one in a field and another in a solar greenhouse 200 m away from the field, at the Shiyanghe Experimental Station of Crop Water Use, Wuwei city of northwest China (37°52′N, 102°50′E, 1581 m above sea level). The field experiment was conducted from May to August 2016 (2016 season) and the greenhouse experiment from April to August 2017 (2017 season). The greenhouse was 76 × 8 m in size and made of a steel frame covered with 0.2-mm thick polyethylene, with no heating or cooling system. A narrow ventilation on the roof controled the interior daytime temperature in the summer.

The pink series tomato (*Lycopersicon esculentum*, cultivars “Nathen” and “Jinpeng”) were grown in the 2016 and 2017 seasons; both are common indeterminate tomato cultivars widely used in local tomato production. During both field and greenhouse experiments, the seedlings were transplanted at the 3rd to 4th leaf stage into 7.8L plastic containers (35 cm top diameter, 30 cm bottom diameter, and 25 cm depth) filled with 16 kg air-dried sandy loam soil (<5 mm) with a bulk density of 1.3 ± 0.5 g cm^−3^. During both years, cheesecloth and 1 kg of small gravel were packed at the bottom of the container to prevent soil loss. The containers were buried in the ground up to the top edge to maintain a soil temperature similar to the field. Soil surface of each container was covered with white polyethylene film to prevent soil water evaporation. The fertilizers applied were 200 mg kg^−1^ soil N (CH_4_N_2_O), 390 mg kg^−1^ soil P (Ca(H_2_PO_4_), and 55 mg kg^−1^ soil K (KH_2_PO_4_). All three fertilizers were mixed homogeneously with soil before filling the containers to support plant growth during both years. The sandy loam soil had an average *in situ* bulk density of 1.52 g cm^−3^, volumetric soil water content of 25.3% at pot water-holding capacity, electrical conductivity of 0.302 dS m^−1^, and pH of 7.88 (Table 1). Considering the cultivars' characteristics of indetermination, tomato plants were pinched when the third truss of flowers came out.

**Table 1:** Mean for some of the physiochemical properties of soil used for field experiment in 2016 and greenhouse experiment in 2017.

**Season**	**Soil texture**	**% Sand**	**% Silt**	**% Clay**	**pH**	**Bulk density**	**Field capacity**	**Soil conductivity**
						**(g cm^**−3**^)**	**(cm^**3**^ cm^**−3**^)**	**(dS m^**−1**^)**
2016	Sandy loam	50	45	5	7.96	1.52	0.258	0.205
2017	Sandy loam	51	45	5	7.8	1.52	0.247	0.398

### Treatments

Two levels of irrigation, full irrigation (W1) and reduced irrigation (W2/3, 2/3 of W1), were applied with four soil salt regimes during two experiments. In the field experiment of 2016, four salt treatments created were S0 (no salt added), S3 (0.3%), S6 (0.6%), and S9 (0.9%), corresponding to the soil solution electrical conductivity (EC_s_) of 0.205, 1.030, 1.932, and 2.597 dS m^−1^. Salt content in parentheses represents the mass ratio of total salts to air-dried soil. Three salts, sodium chloride (NaCl), magnesium sulfate (MgSO_4_), and calcium sulfate (CaSO_4_), were homogeneously mixed with soil prior to packing into containers with the mass ratio of 2:2:1, respectively. In the greenhouse experiment of 2017, S6 and S9 salinity treatments were discontinued and replaced with S2 (0.2%) and S4 (0.4%) with EC_s_ of 0.814, and 1.326 dS m^−1^ because plants were almost dead under S6 and S9 treatments. The experiment design was a split plot with water treatments (two levels) as main plot and salt treatments (four levels) as sub-plot, each treatment has 10 and 20 containers in 2016 and 2017, respectively.

One tomato plant was transplanted to each container at the 3rd to 4th leaf stage on 9 May 2016 and 24 April 2017. Container spacing was 0.5 × 0.4 m, resulting in five plants per m^2^. A drip arrow irrigation system was employed with two-drop arrow emitters in each container. For each treatment, the irrigation volume was controlled by a plastic bucket with scales installed at the head of the drip pipes, and sand and mesh filters were installed to prevent emitter clogging. Tap water with an electrical conductivity of 0.62 dS m^−1^ was used for irrigation. The irrigation treatments started on June 5th in 2016 at flowering stage, and on May 5th in 2017 in the middle of vegetative stage. Tomato plants were irrigated to 90% of pot water-holding capacity for the full irrigation treatment when its average soil water content (observed by 5TE sensors) decreased to 50 ± 2% of pot water-holding capacity. Irrigation amounts and times for each treatment are listed in Table 2.

**Table 2:** Details of irrigation treatment during tomato growth period during 2016–2017.

**Year**	**Growth stage**	**Date (MM/DD)**	**Irrigation amount (L)**		**Irrigation times (No.)**
			**W2/3**	**W1**	
2016	Vegetative	05/09–06/05	10.7	10.7	8
	Flowering	06/06–06/24	9.2	12.5	11
	Fruit development and ripening	06/25–08/11	22.7	32.5	22
	Whole	05/09–08/11	39.7	52.7	41
2017	Vegetative	04/24–05/24	6.9	8.2	8
	Flowering	05/25–06/13	10.1	14.4	10
	Fruit development and ripening	06/14–08/09	30.6	42.8	35
	Whole	04/24–08/09	47.6	62.4	53

*W1, full irrigation; W2/3, deficit irrigation received 2/3 of full irrigation amount; vegetative stage, transplant to the first blossom; flowering stage, first blossom to first fruit set; fruit development and ripening stage, first fruit set to harvesting*.

The entire growth period of tomato was divided into three stages, i.e., vegetative stage (transplant to first blossom), flowering stage (first blossom to first fruit set), and fruit development and ripening stage (first fruit set to harvesting). Details of growth periods and irrigations are shown in Table 2.

### Environmental Variables

**Figure 1:**
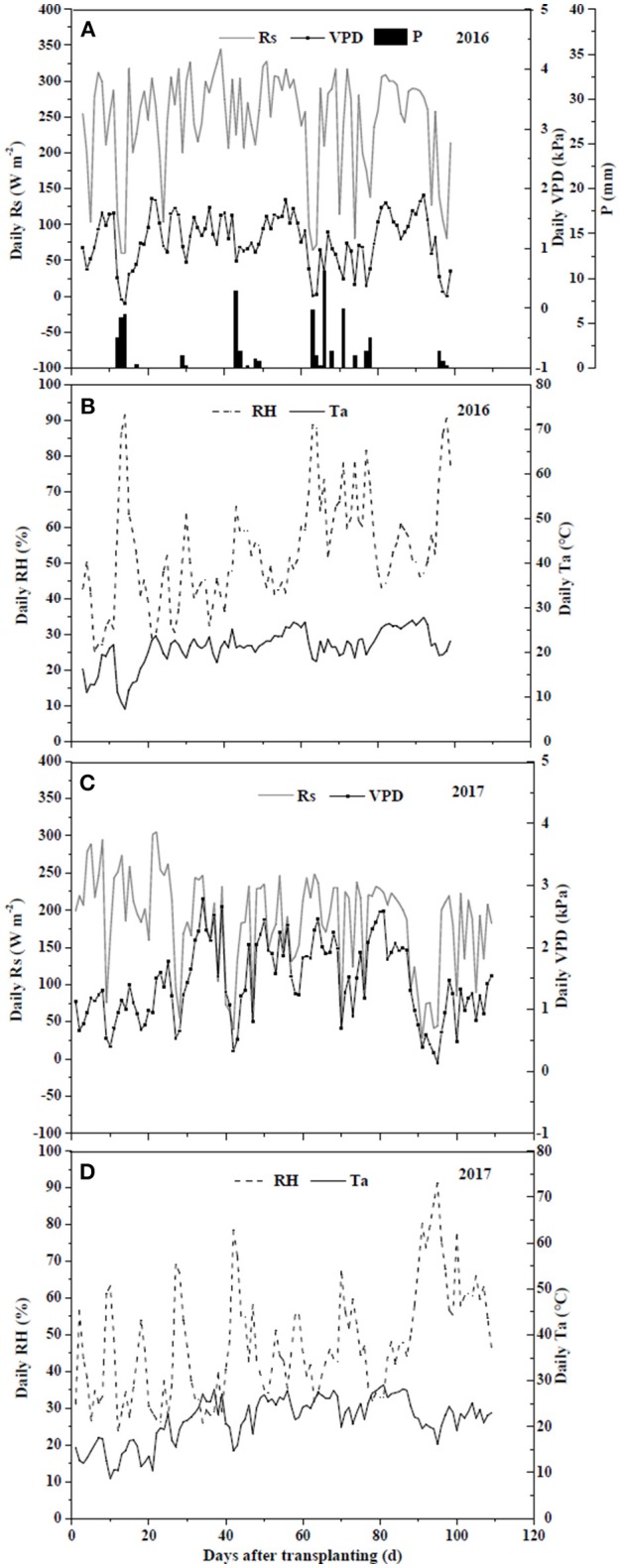
Climate variances including daily solar radiation (R_s_) **(A,C)**, relative humidity (RH) **(B,D)**, temperature (T_a_) **(B,D)**, and vapor pressure deficit (VPD) **(A,C)** of field experiment in 2016 and greenhouse experiment in 2017.

The meteorological factors, solar radiation (R_s_, W m^−2^), relative humidity (RH, %), air temperature (T_a_, °C), and vapor pressure deficit (VPD, kPa), for field and greenhouse experiments are shown in Figure 1. In the field experiment of 2016, meteorological data were recorded every 15 min from a weather station (Weather Hawk, Campbell Scientific, USA) 50 m away from the experiment field. In the greenhouse experiment of 2017, an automatic weather station (HOBO, Onset Computer Corp., USA) was installed in the middle of the greenhouse and data were collected every 15 min. VPD was calculated from RH and T_a_ (Norman, 1998). To measure soil water content (SWC, cm^3^ cm^−3^), one 5TE sensor (Decagon Devices, Inc., USA) was installed at the depth of 15 cm in three randomly selected containers in each treatment in both experiments. The data were collected every 30 min by an EM50 data logger (Decagon Devices, Inc., USA). Sensors were calibrated by optimizing gravimetrically and sensor-measured volumetric water contents.

### Leaf and Plant Measurements

Leaf gas exchange parameters, including photosynthesis rate (P_n_, μmol m^−2^ s^−1^), transpiration rate (T_r_, mmol m^−2^ s^−1^), stomatal conductance (g_s_, mol m^−2^ s^−1^), and the ratio of interacellular CO_2_ concentration (C_i_, μmol CO_2_ mol^−1^) to atmospheric CO_2_ concentration (C_a_, μmol CO_2_ mol^−1^) were determined on fully expanded upper leaves with three replications in each treatment using a Portable Photosynthesis System (LI-6400XT, LI-COR Corporation, USA). Measurements were conducted on random sunny days from 7:00 a.m. to 7:00 p.m. every 2 h on 22 July and 7 August in 2016 and 4 July, 11 July, and 9 August in 2017 at the fruit development and ripening stages. Instantaneous water use efficiency (WUE_ins_, mmol mol^−1^) was defined as the ratio of P_n_ to T_r_ and instrinsic water use efficiency (WUE_int_, μmol mol^−1^) as the ratio of P_n_ to g_s_ (Bierhuizen and Slatyer, 1965; Sinclair et al., 1984).

Sap flow rates of tomato plants were measured using dynagages (SGB9, SGB13, Dynamax, USA) during fruit development and ripening stages with Stem Heat Balance (SHB) method. In this method a stem is wrapped in a heater coil emitting a constant energy flux. The supplied energy is dissipated by convection along the stem by sap flow transport. Therefore, by measuring the convective heat fluxes and the energy supply, the rate of water flux along a stem can be calculated (Trambouze and Voltz, 2001). The dynagages were installed on the stems of plants between the third and fourth internode above the soil surface. Leaf branches beneath the fifth internode were removed and plastic film was placed to avoid stem transpiration. A CR1000 data logger (Campbell Scientific, USA) was used to collect data every 30 s and averaged every 15 min. Consequently, hourly sap flow rate per plant (Q_h_, g h^−1^) was obtained. Since the CR1000 had eight channels, two plants per treatment of W2/3 treatments were first randomly selected to monitor sap flow during 27 June to 6 July, and two plants per treatment of W1 treatments were then selected from 19 July to 28 July in the 2016 season. In 2017, one plant per treatment was selected for sap flow measurement between 25 June and 30 June.

Three tomato plants for each treatment were harvested on 11 August 2016 and 9 August 2017. All fresh fruits from the three plants in each treatment were collected and yield (Y, g per plant) was recorded using an electronic balance with accuracy of 0.01 g (ME2002E, Mettler Toledo, USA). Roots, stems, and leaves were seperately dried at 75 °C in the oven to the constant weight and dry matter weight was recorded. Water use efficiency at plant scale WUE_DM_ was calculated as the ratio of dry aboveground biomass to irrigation water per plant, and WUE_Y_ as fresh yield to irrigation water per plant.

### Statistical Analyses

Two-way analysis of variance was performed using SPSS version 23.0 (IBM Statistics) by year to evaluate the effects of irrigation and salt regimes, as well as their interactions on tomato leaf physiology parameters and WUE at different scales. Duncan's multiple range test was used to assess differences between treatments at *P* = 0.05. Pearson correlation analysis was done for 13 parameters, including WUE_DM_, WUE_Y_, WUE_int_, WUE_ins_, P_n_, T_r_, g_s_, C_i_/C_a_, yield, dry aboveground biomass, sap flow rate, irrigation amount, and SSC (Table 4). The values of leaf physiology parameters were daily averages of all measurements.

Principal component analysis (PCA) was used to evaluate the comprehensive WUE at different scales as affected by reduced irrigation and salt stress regimes. The standardized data included WUE_DM_, WUE_Y_, WUE_int_, and WUE_ins_. PCA was carried out using correlation matrix of SPSS version 23.0 after the KMO and Bartlett's test, and factors with eigenvalues >1 were retained. Using compute variables module in SPSS, PCs were determined and then maximum and minimum principal component for each treatment were calculated. Finally, a comprehensive score was determined for each treatment; the larger the score, the higher the performance of the treatment (Shukla et al., 2006; Wang et al., 2015).

## Results

### Environmental Variables

**Figure 2:**
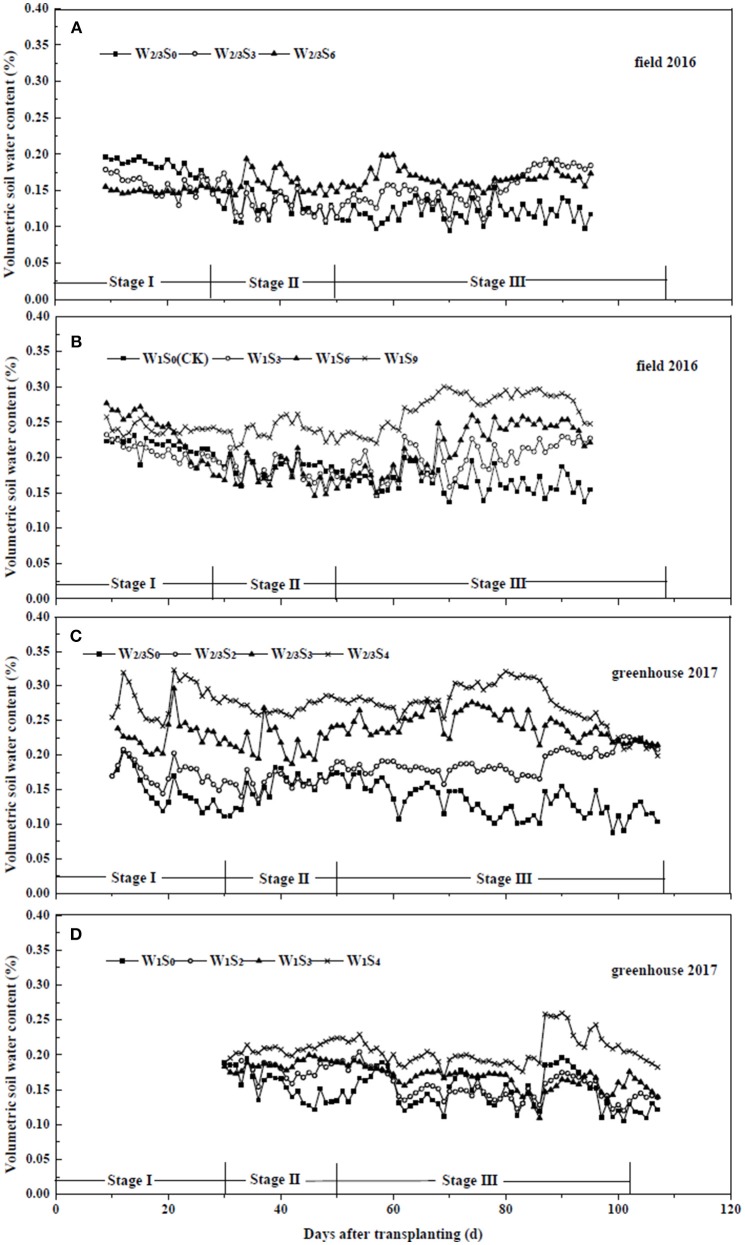
Soil water content of tomato under different water and salt treatments in field experiment of 2016 **(A,B)** and greenhouse experiment of 2017 **(C,D)**. Stage I: vegetative stage, Stage II: flowering stage, Stage III: fruit development and ripening stage.

In the field, R_s_ ranged from 59.7 W m^−2^ on cloudy or rainy days to 327.8 W m^−2^ on sunny days, with an average of 243.1 W m^−2^ (Figure 1). RH, Ta and VPD varied from 25.3 to 91.6%, from 7.2 to 27.8°C, and from 0.08 to 1.89 kPa, with the averages of 53.0%, 21.0°C and 1.14 kPa, respectively. In the greenhouse, R_s_ ranged from 28.2 to 305.1 W m^−2^, with an average of 171.8 W m^−2^. RH, Ta and VPD varied from 23.7 to 91.4%, from 8.7 to 29.1°C, and from 0.14 to 2.78 kPa, with the averages of 50.0%, 23.7°C and 1.56 kPa, respectively. Variations of daily average SWC in the 0–20 cm soil profile under different irrigation and salt stress treatments during field and greenhouse experiments are presented in Figure 2. In the field experiment of 2016, SWC under W1S9 treatment was higher than W1S6, W1S3, and W1S0 treatments during the whole growth season because plants were severely stressed and uptake was very low. Under reduced irrigation, the variation of SWC among S0, S3, and S6 treatments decreased compared to full irrigation (Figures 2A,B). In the greenhouse experiment of 2017, SWC increased with increasing soil salt content under both full and reduced irrigation (Figures 2C,D).

### Yield and Dry Biomass Per Plant

**Figure 3:**
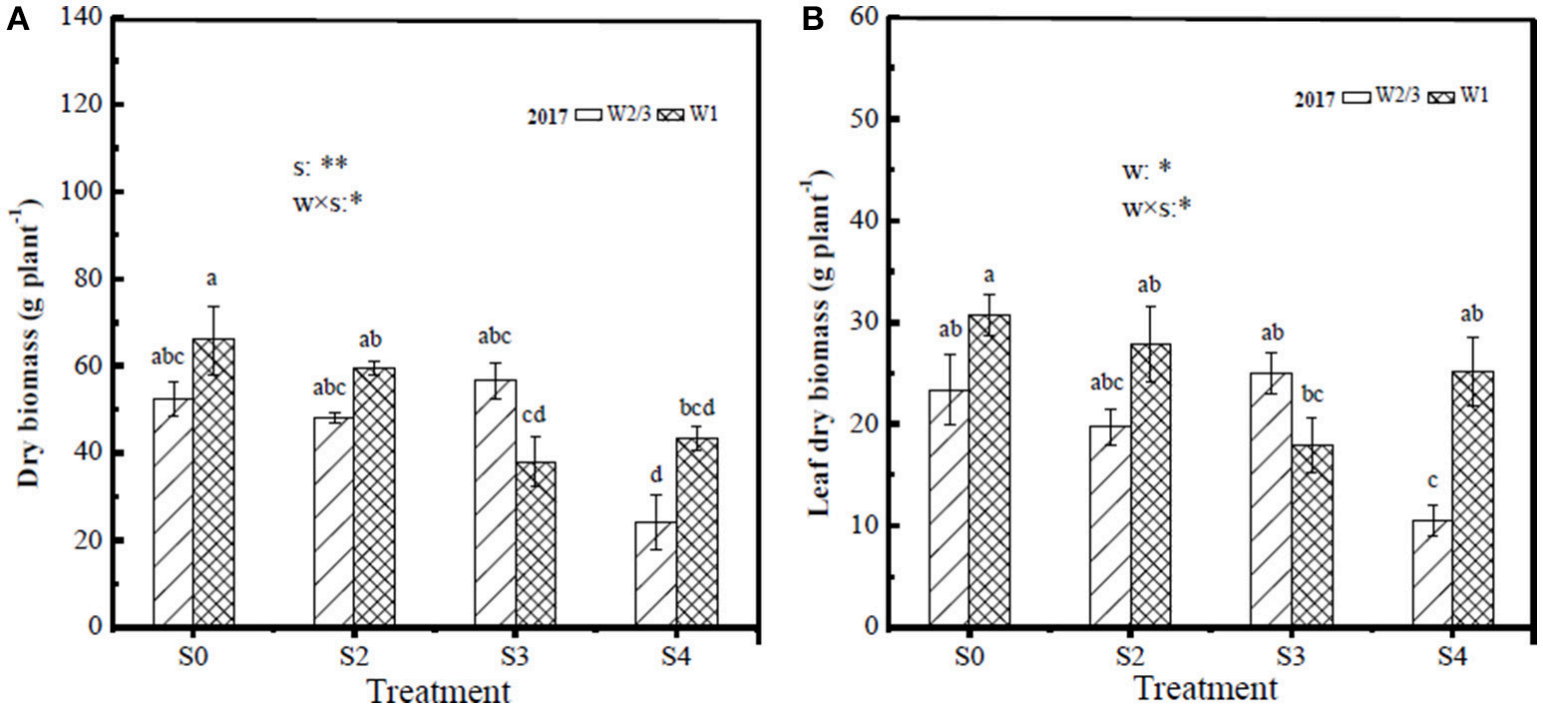
Tomato dry biomass of whole plant **(A)** and leaf dry biomass **(B)** under different water and salt treatments at harvest in 2017. Only the positive effects of the interactions (w × s) are showed in the figure, w, water treatments; s, salt treatments, ^*^significant differences for *P* < 0.05; ^**^significant differences for *P* < 0.01.

In the field experiment of 2016, dry biomass per plant of stem, leaf, root, and total as well as root/shoot ratio were significantly affected by soil salt treatments, while water treatments and the interaction of water and salt had no significant effects. Fresh fruit yield of tomato per plant was influenced by water and salt treatments, and their interaction (Table 3). Increasing soil salt content caused more yield reductions under both irrigation treatments. In the field experiment of 2016, reduced irrigation (W2/3) treatments produced higher yields compared to W1 under S0 and S3 treatments, while W2/3 treatments exacerbated yield reductions under S6 and S9 treatments. In the greenhouse experiment of 2017, since the gradient of soil salt treatments was reduced, salt treatments didnot show significant effects on dry biomass of stem, leaf, root, and root/shoot ratio. Only leaf biomass was affected by water treatments and the interaction of water and salt, and root/shoot ratio was influenced by water treatments. The effect of salt treatments on total biomass per plant was also significant. Yields showed similar trends with those in the field experiment of 2016 under water and salt treatments (Table 3). The proportions of stem, leaf, and root dry matter were about 45.5, 42.9, and 13.4%, respectively (Figure 3).

**Table 3:** P_n_, T_r_, g_s_, C_i_/C_a_, DM, Y, and WUE at different scale of tomato under water and salt treatments at fruit development and ripening stage during 2016–2017.

**Year**	**Treatment**	**Pn (μmol m^**−2**^ s^**−1**^)**	**Tr (mmol m^**−2**^s^**−1**^)**	**gs (mol m^**−2**^s^**−1**^)**	**Ci/Ca**	**WUE_**ins**_ (μmol mmol^**−1**^)**	**WUE_**int**_ (μmol mol^**−1**^)**	**SD (g plant^**−1**^)**	**LD (g plant^**−1**^)**	**RD (g plant^**−1**^)**	**Root/ shoot**	**DM (g plant^**−1**^)**	**Y (g plant^**−1**^)**	**WUE_**DM**_ (g L^**−1**^)**	**WUE_**Y**_ (g L^**−1**^)**
2016	**Water Treatments (w)**														
	W2/3	7.45	4.26	0.090	0.529	1.72	82.3	24.2	21.1	6.8	0.18	50.8	577.0	1.28	14.54
	W1	8.13	4.51	0.102	0.524	1.80	82.7	29.8	22.4	7.4	0.17	55.1	557.3	1.05	10.58
	**Salt Treatments (s)**														
	S0	11.46a	5.96a	0.148a	0.537b	1.93a	77.6d	47.0a	45.7a	10.1a	0.11b	102.7a	1036.4a	2.29a	23.17a
	S3	8.46b	4.95b	0.106b	0.523c	1.72c	80.9b	21.0b	16.9b	7.2b	0.19a	45.1b	753.0b	0.98b	16.64b
	S6	6.49c	3.70c	0.070c	0.481d	1.77b	93.4a	20.5b	16.3b	7.7b	0.21a	41.9b	334.5c	0.91b	7.30c
	S9	4.75d	2.95d	0.061d	0.565a	1.62d	78.3c	9.1c	8.1c	3.6c	0.20a	22.3c	144.7d	0.48c	3.13d
	w	^**^	^**^	^**^	^**^	^**^	^**^	ns	ns	ns	ns	ns	^**^	^**^	^**^
	s	^**^	^**^	^**^	^**^	^**^	^**^	^**^	^**^	^**^	^**^	^**^	^**^	^**^	^**^
	w × s	^**^	^**^	^**^	^**^	^**^	^**^	ns	ns	ns	ns	ns	^**^	^**^	^**^
2017	**Water Treatments (w)**														
	W2/3	10.69	8.91	0.275	0.790	1.21	40.3	18.9	19.7	5.7	0.15	45.4	774.1	0.95	16.26
	W1	11.86	10.31	0.332	0.749	1.16	36.7	20.7	21.5	5.4	0.14	48.8	771.5	0.94	14.90
	**Salt Treatments (s)**														
	S0	10.97b	9.55c	0.300c	0.865a	1.18a	39.4b	26.1a	27.1a	6.1a	0.12a	59.2a	1052.6a	1.08a	19.50a
	S2	12.11a	11.01a	0.353a	0.743c	1.12b	35.1d	21.5a	23.8a	6.4a	0.14a	54.0a	787.7b	0.98b	14.64b
	S3	11.01b	9.90b	0.309b	0.731d	1.11b	36.9c	20.4a	21.6a	5.4a	0.13a	47.4ab	694.2c	0.90ab	12.90c
	S4	9.08c	8.59d	0.228d	0.750b	1.06c	39.8a	16.6a	17.8a	4.7a	0.14a	33.8b	526.2d	0.61b	9.73d
	w	^**^	^**^	^**^	^**^	^**^	^**^	ns	^*^	ns	^*^	ns	^*^	ns	^**^
	s	^**^	^**^	^**^	^**^	^**^	^**^	ns	ns	ns	ns	^**^	^**^	^**^	^**^
	w × s	^**^	^**^	^**^	^**^	^**^	^**^	ns	^*^	ns	ns	^*^	ns	^*^	^**^

*P_n_, photosynthetic rate; T_r_, transpiration rate; g_s_, stomatal conductance; C_i_, intercellular CO_2_ concentration; C_a_, atmospheric CO_2_ concentration; WUE_ins_, instantaneous water use efficiency at leaf scale; WUE_int_, intrinsic water use efficiency at leaf scale; SD, stem biomass per plant; LD, leaf biomass per plant; RD, root biomass per plant; DM, dry aboveground biomass per plant; Y, fresh yield per plant; WUE_DM_, water use efficiency at dry biomass level; WUE_Y_, water use efficiency at yield scale. The values are the averages of the two water treatments W2/3 and W1 and the same for salt treatments (S0, S3, S6, and S9). The gas exchange values (except DM, Y, WUE_DM_, and WUE_Y_) represent the daily mean of sample measurements on 07/22 and 08/07 in 2016 and 07/04, 07/11, and 08/09 in 2017. Differences due to treatments were determined using analyses of variance for each season, w, water effect; s, salt effect; w × s, interactive effect of water and salt*.

* Significant differences for P < 0.05;

** *Significant differences for P < 0.01; ns, no significant*.

### Gas Exchange Parameters

All the gas exchange parameters P_n_, T_r_, g_s_, and C_i_/C_a_ were significantly affected by irrigation regimes, salt treatment, and their interactions in both experiments (Table 3). In 2016, P_n_ and T_r_ decreased with increasing soil salt stress under both W2/3 and W1 treatments. However, in the greenhouse experiment of 2017, plants grown in salt treatments of S2 and S3 had notably 35.5 and 49.4% higher P_n_, respectively, than those grown in S0 treatment under reduced irrigation regime (W2/3). For W1 treatment, highest P_n_ of 13.30 μmol m^−2^ s^−1^ was for S0 treatment (Table 3). Moreover, T_r_ was significantly positively correlated with P_n_ and g_s_ and was also notably correlated with P_n_ and T_r_ (Table 4). Although irrigation amount and soil salt content notably affected C_i_/C_a_ (Table 3), Pearson correlation coefficients between C_i_/C_a_, irrigation amount and soil salt content were not significant; C_i_/C_a_ had significant positive correlation with T_r_ and g_s_ (Table 4).

**Table 4:** Pearson correlation coefficients between WUE at leaf and plant scales and irrigation amount, soil salt content, sap flow rate, leaf physiological parameters, dry matter per plant, and fresh yield per plant.

**Index**	**WUE_**ins**_ (μmol mmol^**-1**^)**	**WUE_**int**_ (μmol mol^**-1**^)**	**WUE_**DM**_ (g L^**−1**^)**	**WUE_**Y**_ (g L^**-1**^)**	**I (L)**	**SSC (%)**	**P_**n**_ (μmol m^**−2**^ s^**−1**^)**	**T_**r**_ (mmol m^**−2**^ s^**−1**^)**	**g_**s**_ (mol m^**−2**^ s^**−1**^)**	**C_**i**_/C_**a**_**	**DM (g plant^−1^)**	**Y (g plant^**−1**^)**
WUE_int_ (μmol mol^−1^)	0.934^**^											
WUE_DM_ (g L^−1^)	0.454	0.185										
WUE_Y_ (g L^−1^)	0.130	−0.139	0.803^**^									
Irrigation amount (L)	−0.569^*^	−0.558^*^	−0.265	−0.192								
Soil salt content (%)	0.169	0.423	−0.639^**^	−0.882^**^	−0.213							
P_n_ (μmol m^−2^ s^−1^)	−0.390	−0.648^**^	−0.467	0.620^*^	0.365	−0.827^**^						
T_r_ (mmol m^−2^ s^−1^)	−0.801^**^	−0.918^**^	0.030	0.279	0.597^*^	−0.586^*^	0.850^**^					
g_s_ (mol m^−2^ s^−1^)	−0.766^**^	−0.896^**^	0.033	0.260	0.543^*^	−0.540^*^	0.846^**^	0.986^**^				
C_i_/C_a_	−0.841^**^	−0.892^**^	−0.190	0.206	0.417	−0.430	0.462	0.730^**^	0.716^**^			
DM (g plant^−1^)	0.298	0.011	0.939^**^	0.764^**^	0.047	−0.752^**^	0.638^**^	0.239	0.233	−0.055		
Y (g plant^−1^)	−0.098	−0.366	0.686^**^	0.930^**^	0.150	−0.981^**^	0.782^**^	0.521^*^	0.490	0.393	0.771^**^	
Sap flow rate (g h^−1^)	0.175	−0.042	0.480	0.396	0.489	−0.577^*^	0.526	0.258	0.252	0.031	0.684^**^	0.581^*^

*P_n_, photosynthetic rate; T_r_, transpiration rate; g_s_, stomatal conductance; C_i_, intracellular CO_2_ concentration; C_a_, atmospheric CO_2_ concentration; I, irrigation amount; SSC, soil salt content; DM, dry aboveground biomass per plant; Y, fresh yield per plant; WUE_ins_, instantaneous water use efficiency at leaf scale; WUE_int_, intrinsic water use efficiency at leaf scale; WUE_DM_, water use efficiency at dry biomass level; WUE_Y_, water use efficiency at yield scale. The values of all parameters used for analyzing were daily averages (except I, SSC, DM, and Y) collected during 2016–2017*.

* Significant differences for P < 0.05;

** *Significant differences for P < 0.01*.

**Figure 4:**
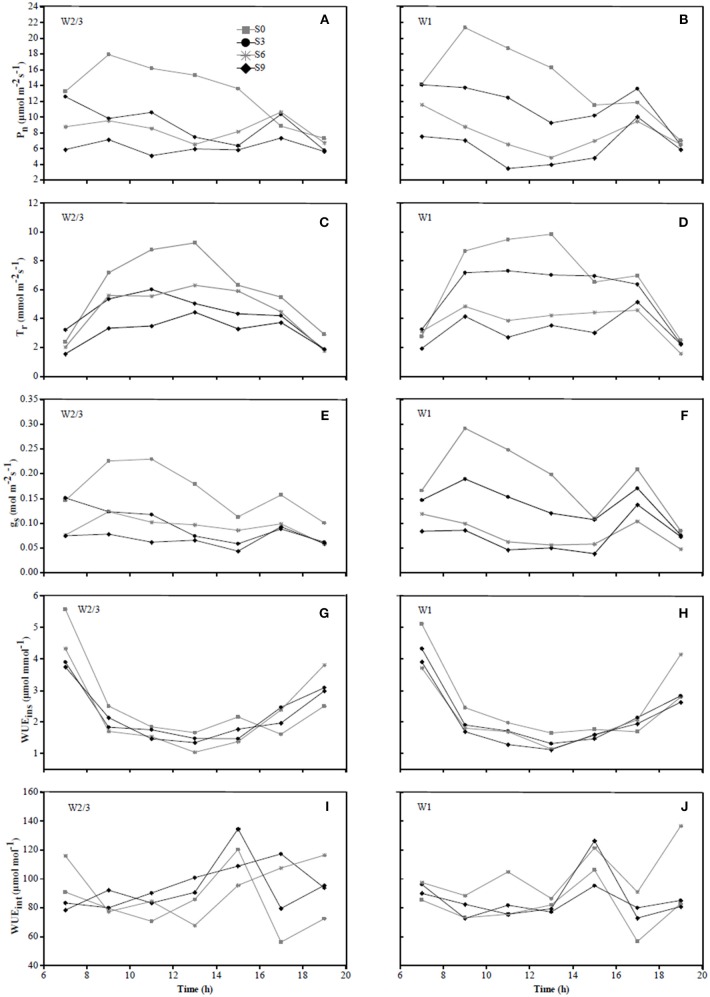
Diurnal variations in photosynthetic rate (P_n_) **(A,B)**, transpiration rate (T_r_) **(C,D)**, stomatal conductance (g_s_) **(E,F)**, instantaneous water use efficiency (WUE_ins_) **(G,H)**, and intrinsic water use efficiency (WUE_int_) **(I,J)** of tomato under different water treatments at fruit ripening stage (date 07/22) in 2016.

**Figure 5:**
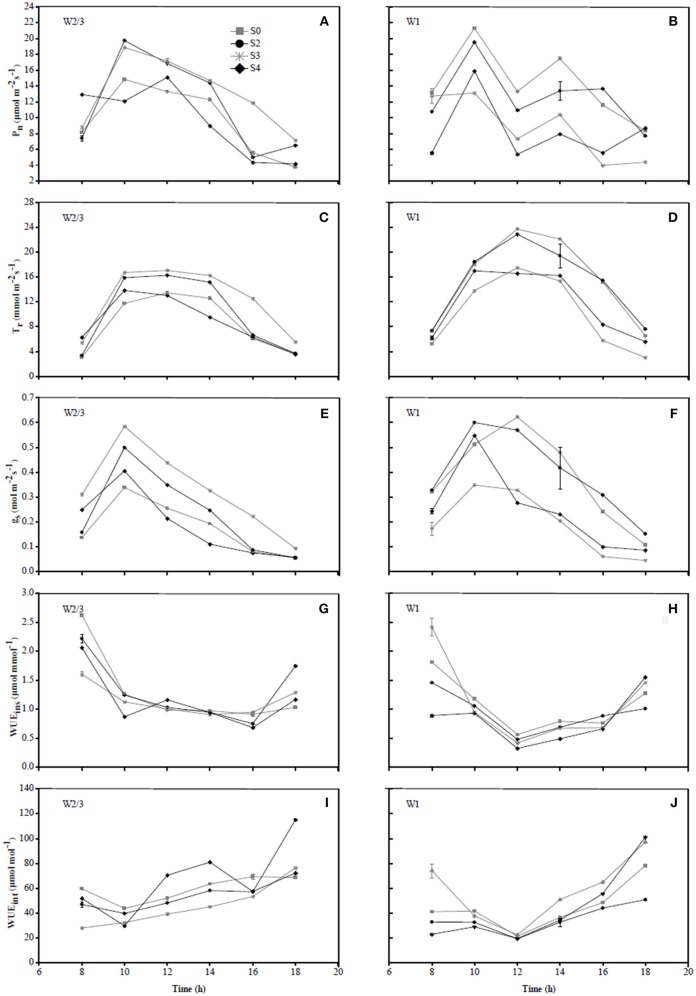
Diurnal variations in photosynthetic rate (P_n_) **(A,B)**, transpiration rate (T_r_) **(C,D)**, stomatal conductance (g_s_) **(E,F)**, instantaneous water use efficiency (WUE_ins_) **(G,H)**, and intrinsic water use efficiency (WUE_int_) **(I,J)** of tomato under different water treatments at fruit ripening stage (date 07/11) in 2017.

In 2016, diurnal variations of P_n_ and g_s_ in S0 treatment under W2/3 and W1 regimes initially showed an increase from 7:00 to 9:00 a.m. and a decrease until 3:00 p.m.; however, multiple peaks were observed around 5:00 p.m., compensation of mild salt stress (S3) in P_n_ was more obvious than that of S0 (Figures 4A,B,E, F). In 2017, the diurnal variations of g_s_ under various water and salt treatments showed a single-peak curve with the peak occurring at 10:00 a.m. (Figures 5E,F), and T_r_ remained relatively high between 10:00 a.m. and 2:00 p.m. (Figures 5C,D), which was consistent with the results in the field experiment during 2016 (Figures 4C,D).

### Sap Flow Variation

**Figure 6:**
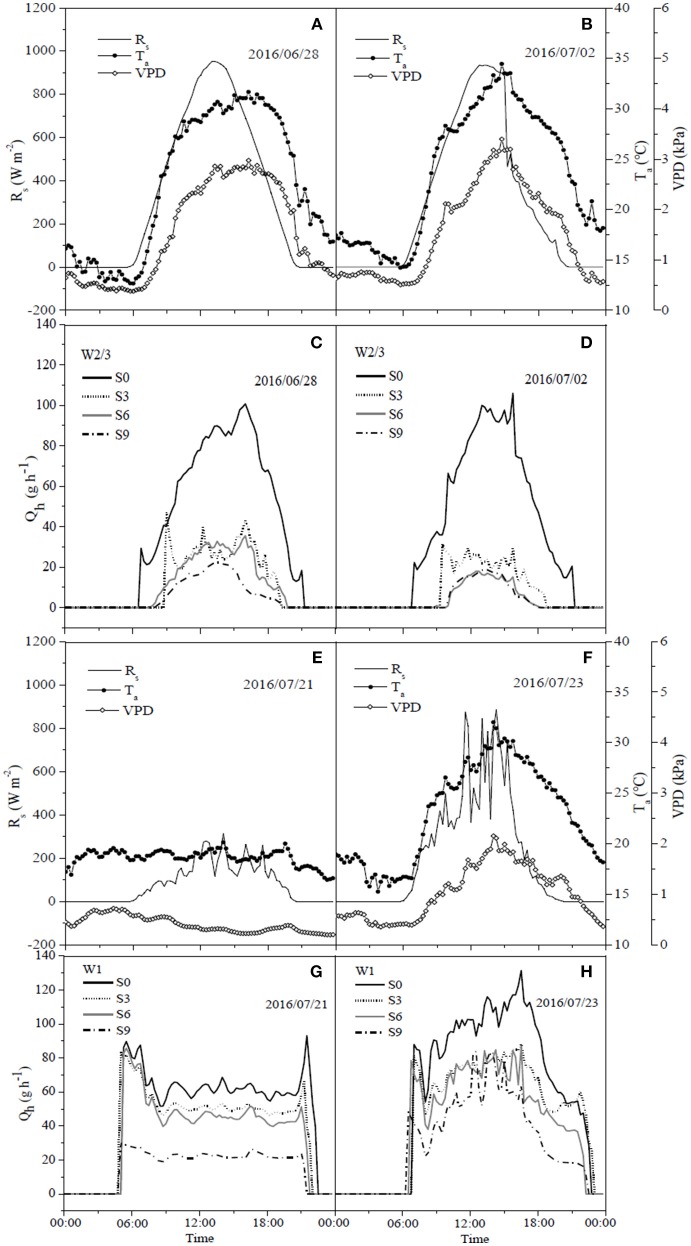
Diurnal dynamics of hourly sap flow rate per plant (Q_h_) **(C,D,G,H)** in tomato and corresponding solar radiation (R_s_), air temperature (T_a_) and vapor pressure deficit (VPD) **(A,B,E,F)** under different water and salt treatments in 2016.

**Figure 7:**
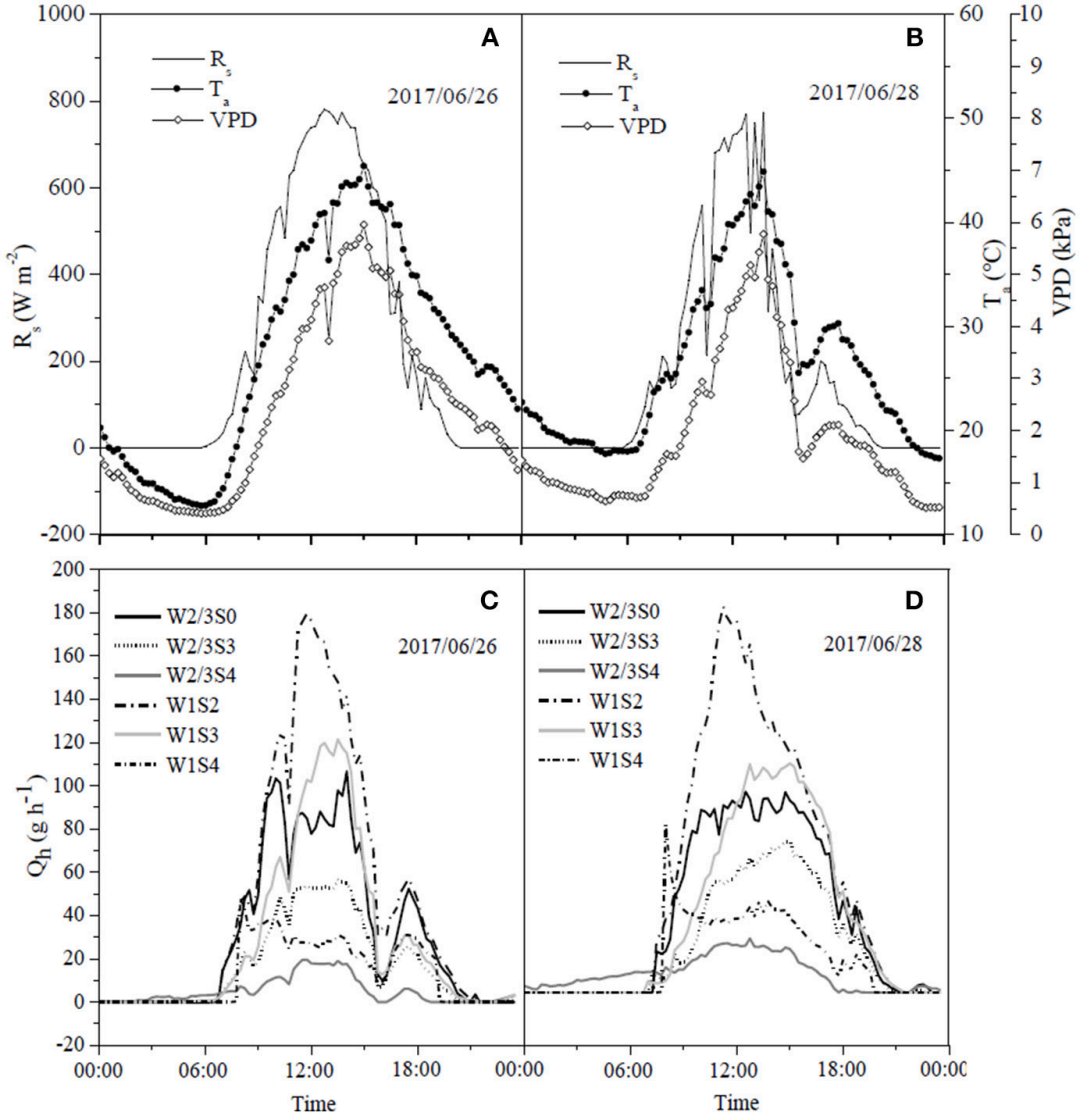
Diurnal dynamics of hourly sap flow rate per plant (Q_h_) **(C,D)** in tomato and corresponding solar radiation (R_s_), air temperature (T_a_), and vapor pressure deficit (VPD) **(A,B)** under different water and salt treatments in 2017.

The daily variations of sap flow rate per plant under irrigation and salt treatments varied diurnally. Sap flow increased significantly from early morning (7:00 a.m.), reached the maximum around noon, but notably decreased after 4:00 p.m., with no sap flow measured at 9:00 p.m. (Figures 6, 7) in both experiments. In the field experiment, increases in sap flow in the morning were significantly delayed with increasing soil salt content, maximum sap flow was much lower than that in S0, and sap flow stopped earlier in the night (Figures 6C,D). The diurnal variations in sap flow under various water and salt treatments showed a single-peak curve, and sap flow rate was smaller throughout the day with increasing soil salt content under both W2/3 and W1 treatments (Figures 6C,D,G,H). In the greenhouse experiment, however, diurnal variations in sap flow showed a peak around noon, and another small peak was observed at 6:00 p.m. W1S2 treatment showed a relatively higher sap flow throughout the day, following the W1S3 and W2/3S0 treatments. Soil salt content significantly decreased daily sap flow (Table 4). The daily variation of sap flow rate per plant ranged from 7.96 to 31.37 g h^−1^ in the field experiment and from 12.52 to 36.02 g h^−1^ in the greenhouse experiment among various salt stresses.

The relationships between sap flow rates and climate variables under different irrigation and salt treatments at fruit development and ripening stage were established in the field and greenhouse experiments. The significant level of regression coefficients was less than 0.01, and the most of correlation coefficients (*R*^2^) were higher than 0.5 (Table 5). In 2016, sap flow rate under W2/3 treatments increased linearly with increasing R_s_, T_a_, and VPD (*R*^2^ > 0.5). In 2017, sap flow also showed a positive correlation with R_s_, T_a_, and VPD under various water and salt treatments except W2/3S4 treatment (*R*^2^ = 0.41 for T_a_ and 0.46 for VPD) (Table 5). The different regression equations indicated that the sensitivity of sap flow rates to climate variables varied with the irrigation regimes and salt treatments. In both experiments, the slopes of regression equations in no salt treatments (W2/3S0 and W1S0) were higher than those in salt stress treatments. The average *R*^2^ between T_a_ and VPD were lower due to the lagging effects on sap flow rates (Figures 6, 7). The climate variables affecting sap flow rates were ranked as R_s_ > VPD>T_a_.

**Table 5:** Relationships between sap flow rates (Q_h_, g h^−1^) every 15 min and corresponding solar radiation (R_s_, W m^−2^), air temperature (T_a_, °C), and vapor pressure (VPD, kPa) deficit under different irrigation and salt stress treatments during 2016 and 2017 seasons.

**Growth season**	**Treatment**	**N**	**R**_****s****_		**T**_****a****_		**VPD**	
			**Regression equation**	***R*^**2**^**	**Regression equation**	***R*^**2**^**	**Regression equation**	***R*^**2**^**
2016	W2/3S0	192	Q_h_ = (0.097 ± 0.003)R_s_+5.63	0.86	Q_h_ = (4.91 ± 0.16)T_a_-77.25	0.83	Q_h_ = (36.49 ± 0.98)VPD−20.31	0.88
	W2/3S3	192	Q_h_ = (0.032 ± 0.001)R_s_-0.26	0.76	Q_h_ = (1.47 ± 0.09)T_a_-23.99	0.59	Q_h_ = (11.03 ± 0.53)VPD−7.671	0.70
	W2/3S6	192	Q_h_ = (0.026 ± 0.001)R_s_-0.92	0.76	Q_h_ = (1.08 ± 0.08)T_a_-17.80	0.49	Q_h_ = (8.44 ± 0.52)VPD−5.86	0.58
	W2/3S9	192	Q_h_ = (0.019 ± 0.001)R_s_-1.112	0.87	Q_h_ = (0.753 ± 0.05)T_a_-12.5	0.51	Q_h_ = (5.83 ± 0.35)VPD−4.14	0.59
	W1S0	96	Q_h_ = (0.048 ± 0.01)R_s_+36.5	0.16	Q_h_ = (3.29 ± 0.54)T_a_-29.65	0.28	Q_h_ = (28.2 ± 5.04)VPD+17.21	0.24
	W1S3	96	Q_h_ = (0.039 ± 0.01)R_s_+30.24	0.14	Q_h_ = (2.42 ± 0.49)T_a_-17.54	0.20	Q_h_ = (20.54 ± 4.55)VPD+17.06	0.17
	W1S6	96	Q_h_ = (0.037 ± 0.01)R_s_+26.35	0.14	Q_h_ = (2.19 ± 0.47)T_a_-16.59	0.18	Q_h_ = (18.49 ± 4.41)VPD+14.87	0.15
	W1S9	96	Q_h_ = (1.262 ± 0.19)R_s_-13.22	0.31	Q_h_ = (1.26 ± 0.19)T_a_-13.2	0.31	Q_h_ = (10.81 ± 1.79)VPD+4.71	0.27
2017	W2/3S0	192	Q_h_ = (0.127 ± 0.004)R_s_+6.53	0.86	Q_h_ = (3.50 ± 0.13)T_a_-61.12	0.78	Q_h_ = (19.97 ± 0.87)VPD−10.99	0.73
	W2/3S3	192	Q_h_ = (0.08 ± 0.002)R_s_+2.84	0.88	Q_h_ = (2.31 ± 0.07)T_a_-42.59	0.86	Q_h_ = (13.72 ± 0.38)VPD−10.52	0.87
	W2/3S4	192	Q_h_ = (0.024 ± 0.001)R_s_+1.94	0.83	Q_h_ = (0.50 ± 0.04)T_a_-6.44	0.41	Q_h_ = (3.13 ± 0.25)VPD−10.15	0.46
	W1S2	192	Q_h_ = (0.209 ± 0.005)R_s_+5.69	0.91	Q_h_ = (5.45 ± 0.24)T_a_-97.5	0.74	Q_h_ = (31.25 ± 1.48)VPD−19.56	0.70
	W1S3	192	Q_h_ = (0.137 ± 0.004)R_s_+3.13	0.86	Q_h_ = (3.96 ± 0.12)T_a_−74.82	0.86	Q_h_ = (23.81 ± 0.59)VPD−20.75	0.90
	W1S4	192	Q_h_ = (0.049 ± 0.002)R_s_+3.63	0.69	Q_h_ = (1.30 ± 0.08)T_a_-21.32	0.58	Q_h_ = (7.08 ± 0.51)VPD−1.97	0.50

*N, sample number; the significance of regression coefficients is less than 0.01*.

### Water Use Efficiency (WUE) at Leaf and Plant Scales

Irrigation and soil salt treatments, and their interactive effects on tomato WUE_ins_ and WUE_int_ at leaf scale, were significant during both field and greenhouse experiments (Table 4). In the field experiment of 2016, WUE_ins_ ranged from 1.50 to 1.95 μmol mmol^−1^, while WUE_int_ ranged from 74.5 to 98.9 μmol mol^−1^ under different water and salt treatments. Under full and reduced irrigation, increasing salt stress decreased P_n_ and T_r_ and led to the reductions in WUE_ins_ at the leaf scale. However, compared to full irrigation, reduced irrigation (W2/3) improved WUE_ins_ with a significant decline in g_s_ and T_r_ under no salt and 0.3% soil salt treatments. Under soil salt content of 0.6% and 0.9%, a slight increase in P_n_ and decline in T_r_ resulted in higher WUE_ins_ in the full irrigation treatment. Significant reduction in g_s_ led to increasing WUE_int_ under S3 and S6 treatments compared to S0 treatment under both irrigations; however, further increases in soil salt content to 0.9% decreased the WUE_int_.

In the greenhouse experiment of 2017, WUE_ins_ and WUE_int_ varied from 0.95 to 1.28 μmol mmol^−1^ and from 30.3 to 46.7 μmol mol^−1^, respectively. Compared to full irrigation, WUE_int_ under reduced irrigation was higher under no salt and 0.2% soil salt treatment in 2016, and WUE_DM_ and WUE_Y_ of tomato at whole plant scale were also significantly affected by irrigation regime, soil salt content, and their interaction in 2016 (Table 4), while in 2017, the effect of irrigation regime on WUE_DM_ was not significant. In the field experiment of 2016, increasing soil salt content decreased plant dry biomass and yield, and resulted in greater decreases in whole plant WUE_DM_ and WUE_Y_ under both irrigation treatments. Compared to full irrigation, reduced irrigation increased both WUE_DM_ and WUE_Y_ under 0 and 0.3% soil salt treatments. In the greenhouse experiment of 2017, WUE_DM_ in W2/3S4 treatment was significantly lower than that in W2/3S0 treatment. WUE_Y_ decreased with increasing soil salt content under both irrigation treatments. WUE_Y_ under reduced irrigation was higher compared to full irrigation under 0, 0.2, 0.3, and 0.4% soil salt treatments.

### PCA for Comprehensive Evaluation of WUE

**Figure 8:**
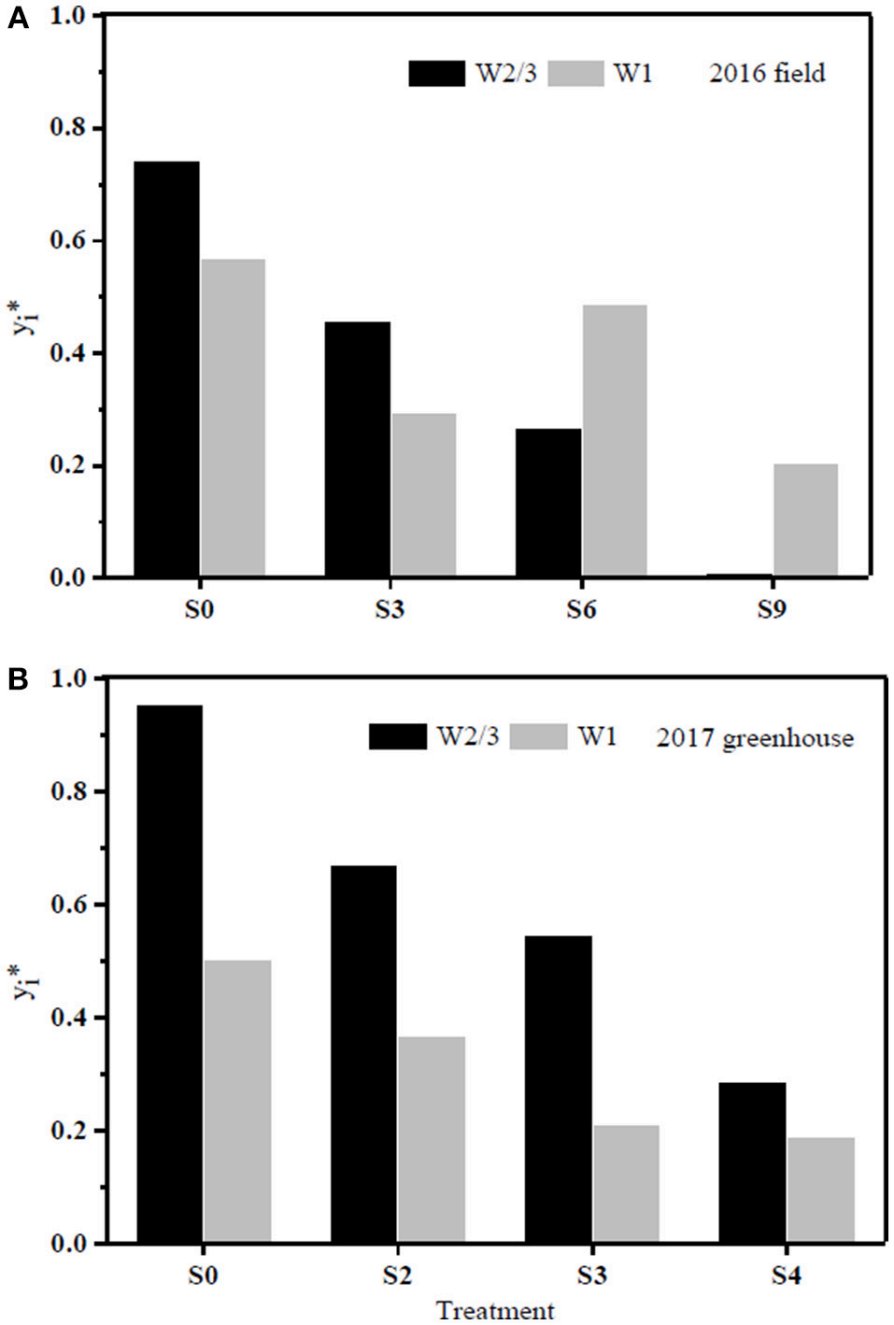
PCA evaluation of tomato water use efficiency (WUE) at leaf and plant scales (four parameters: WUE_ins_, WUE_int_, WUE_DM_, and WUE_Y_) among all the treatments during 2016 **(A)** and 2017 **(B)**, respectively. y${}_{\text{i}}^{*}$ represents closeness of principal component of each treatment to the maximum principal.

The results of PCA evaluation of tomato WUE at leaf and plant scales among all the treatments in the field and greenhouse experiments are shown in Figure 8. The index y${}_{\text{i}}^{*}$ represents closeness of principal component of the treatment to the maximum principal; the larger the index value, the better the performance of the treatment. The PCA analysis showed that plants grown under both full and reduced irrigation with zero soil salt content (W2/3S0 and W1S0) had optimal comprehensive WUE with the highest y${}_{\text{i}}^{*}$ values of 0.743 and 0.569 in the field experiment of 2016. W2/3S0 and W2/3S2 treatments ranked first and second in the greenhouse experiment of 2017, with y${}_{\text{i}}^{*}$ values of 0.953 and 0.670, respectively.

## Discussion

It has been suggested that high soil salt content reduces plant water uptake (Reina-Sánchez et al., 2005; Machado and Serralheiro, 2017; Phogat et al., 2018), which supported our result where root zone soil water content remained higher in containers with higher soil salt content in both reduced and full irrigation treatments during both years (Figure 2). Our results were also consistent with Reina-Sánchez et al. (2005) who reported that tomato plants grown under 75 mM NaCl consumed 40% less water than plants under non-saline condition. Several mechanisms are responsible for this decrease, including modulation of underlying growth mechanisms. Saline growth medium adversely affects plant growth due to low soil osmotic potential (high osmotic stress), resulting in lower leaf and root water potentials, relative water content, and plant dehydration (Ashraf, 2004; Maggio et al., 2004). Salinity-induced ion toxicity, macro and micro nutrient deficiency (such as N, Ca, K, P, Fe, and Zn), as well as oxidative stresses on plants also limit water uptake from soil (Shrivastava and Kumar, 2015).

In this study, dry aboveground tomato biomass per plant was prominently affected by soil salt content, while the effect of irrigation was non-significant in both field and greenhouse experiments (Table 3). These results contrasted with those of Álvarez et al. (2018). A likely explanation is that the Álvarez et al. (2018) experiments were conducted under simulated conditions of soil cultivation and were irrigated with different levels of saline solutions. Uptake and transport rates of saline ions from root to shoot differ when plants are subjected to nutritive solution and multiple salts (Albaladejo et al., 2017) due to the competition among ions (i.e., K^+^, Ca^2+^, NO${}_{3}^{-}$ Na^+^, Cl^−^, and SO${}_{4}^{2}-$) (Hu and Schmidhalter, 2005; Hussain et al., 2016). Most vegetable crops, including tomato, had a salinity threshold of 2.5 dS m^−1^ (Machado and Serralheiro, 2017). However, in this study, 32.9 and 20.7% yield reductions were observed at the EC of 1.03 dS m^−1^ for soil salt content of 0.3% under 2/3 of full irrigation and full irrigation, respectively, in the field experiment, and 23.6 and 26.7% at the EC of 0.81 dS m^−1^ for soil salt content of 0.2% under 2/3 of full irrigation and full irrigation, respectively, in the greenhouse experiment. Thus, the salinity threshold in this study was at or below 0.81 dS m^−1^. This difference could result from species and different saline ions in solution and in the soil, suggesting that multiple salts in soil could further aggravate the adverse effect of salt stress on plants.

Previous studies have observed that reduced irrigation could enhance plant water use efficiency because of only slight decreases in yield but moderate declines in water application as compared to full irrigation (Kang et al., 2002, 2017; Du et al., 2010; Patanè et al., 2011; Yang et al., 2017). This study clearly illustrated that reduced irrigation improved plant yield and dry biomass WUE (WUE_Y_ and WUE_DM_) under low soil salt content (SSC ≤ 0.3%) in both experiments (Table 3). However, in 2016, WUE_Y_ and WUE_DM_ decreased with further increases in soil salt content (SSC ≥ 0.9%) due to the significant reduction of yield and dry biomass. Khataar et al. (2018) results for wheat WUE were similar to this study under low salinities (EC ≤ 8 dS m^−1^). However, Khataar et al. (2018) reported that bean WUE increased with increasing water and salt stresses and contradicted our results for tomato at higher soil salt content.

Tomato P_n_, yield, dry biomass, WUE_Y_, and WUE_DM_ decreased with increasing soil salt content under both full and reduced irrigation during the field experiment (Table 3), indicating the source-sink relationships in which the source organ (leaves) gained the assimilation product through photosynthesis and delivered it to the sink organ (fruits) (Liu et al., 2017). Inverse relationships between P_n_ and increasing salt stress are also reported by other researchers (Netondo et al., 2004; Chaves et al., 2009; Senguttuvel et al., 2014; Das et al., 2015; Negrão et al., 2017).

Stomata control, both water losses and CO_2_ assimilation, is a vital mechnism for plant acclimation to varying environments. Abiotic stress could suppress cell growth and photosynthesis (Wilkinson and Davies, 2002; Liu et al., 2006; Zhang et al., 2015; Álvarez et al., 2018). On the other hand, Galmés et al. (2007) reported that limited recovery of leaf hydraulic conductivity of some species after re-watering could also cause down-regulation of stomatal conductance. In addition, sodium, which was added to soil in this study, disturbs stomatal regulation by interfering with potassium uptake and transport (Farooq et al., 2015). Photosynthesis is affected by changes in stomatal conductance through the pathways noted above. All these are in good agreement with the present study, which found that g_s_ was significantly positively correlated to P_n_ as well as T_r_ (Table 4) under various water and salinity conditions.

Wei et al. (2018) reported that reduced irrigation improved WUE_ins_ and WUE_int_ of tomato with significant decrease in gs and Tr at leaf scale. However, the effects of salt stress and the interaction of water and salt stresses on tomato WUE at leaf scale are still unknown. In the present study, salt stress reduced WUE_ins_ under both irrigation treatments; WUE_ins_ and WUE_int_ improved under reduced irrigation compared to full ittigation when soil salt content was less than 0.4%. Both water and salt treatments together with their interaction had significant effects on WUE_ins_ and WUE_int_ in both experiments (Table 3). In the field experiment of 2016, WUE_ins_ and WUE_int_ of tomato increased under S6 and S9 salt treatments compared to S0 and S3 treatments only when full irrigation was applied, indicating that g_s_ and T_r_ were more sensitive to drought and salinity than P_n_. Diurnal variation of WUE_ins_ in Figures 4, 5 showed that WUE_ins_ in various water regimes and soil salt contents were relatively high in the morning and late afternoon, while it remained low from 10:00 a.m. until 4:00 p.m. during both years. In contrast, diurnal variation of WUE_int_ remained inconsistent during 2016 as well as 2017. The ratio of C_i_ to C_a_ is characteristically near 0.7 for non-stressed C_3_ plants (Farquhar et al., 1989). Lower C_i_:C_a_ ratios resulting from either lower stomatal conductance or higher photosynthetic capacity could improve plant WUE_int_ (Condon et al., 2002). Our study found that the value of C_i_:C_a_ ranged from 0.5 to 0.6 in 2016, whereas it was near 0.7 in 2017 (except W2/3S0 treatment) (Table 3). Lower C_i_:C_a_ ratios were in accord with positive correlations with T_r_ and g_s_.

Liu et al. (2010) reported that on sunny days sap flow showed a single-peaked curve starting at 6:00 a.m., rapidly increased to the maximum at 1:00 p.m. with increasing R_s_ and VPD, and decreased after 2:00 p.m. On cloudy days, sap flow exhibited a multimodal curve corresponding to the variation of R_s_, and there was no flow during the night. In our field experiment, the sap flow also began at 6:00 a.m., but the maximum was reached at around 3:00 p.m. (Figures 6C,D). Differences in these studies are mostly due to the different atmospheric conditions (R_s_, VPD, and T_a_). In the greenhouse experiment, sap flow exhibited a double-peaked curve with the maximum around 12:00 a.m., and the second small peak appeared at 5:30 p.m. (Figures 7C,D), which was affected by the variation of daily R_s_, T_a_, and VPD (Figures 7A,B). R_s_, VPD, and T_a_ had positive linear relationships with sap flow, and the three variables affecting sap flow were ranked as R_s_ >VPD>T_a_ (Table 5) in both experiments, which was in good agreement with Liu et al. (2010) for tomato and Jiang et al. (2016) for maize.

The sap flow in our study also showed a multimodal curve on cloudy days (Figure 6G). It has been reported that reduced irrigation restricted tomato sap flow (Liu et al., 2010; Qiu et al., 2015; Mao et al., 2017), whereas our study found that sap flow was significantly related to soil salt content, and salt stress prominently decreased sap flow rate and delayed start in the morning (Table 4 and Figures 6C,D). The daily variation of sap flow was possibly caused by decreasing plant size and leaf area with increasing salt stress. This result was supported by the significant positive Pearson correlation between daily sap flow rate, dry matter, and fresh yield per plant (Table 4). PCA approach is an effective tool for simplifying data sources, which can realize the accurate and comprehensive assessment of variance source after the feature extraction and dimensionality reduction. In the present study, the PCA appraisement of integrated WUE attributes of tomato grown under water and salt stress included four parameters (WUE_ins_ and WUE_int_ at leaf level and WUE_Y_ and WUE_DM_ at plant level). Reduced irrigation in the absence of salt stress (W2/3S0 treatments) achieved the highest comprehensive WUE among all the treatments both in 2016 and 2017; in addition, 2/3 of full irrigation combined with mild salt stress (soil salt content of 0.2%) showed a relative higher integrated WUE in 2017 (Figure 8).

## Conclusion

This study is important for regulating water-saving strategies for saline soil environments and improving water use efficiency at various scales 2/3 of full irrigation coupled with low soil salt contents (SSC ≤ 0.3%) improved WUE_Y_ and WUE_DM_ at the plant level, but WUE_Y_ and WUE_DM_ decreased under the salt stress of 0.6 and 0.9%. Plants irrigated with full irrigation compensated for salt stress and maintained yield and dry biomass. 2/3 of full irrigation in the absence of salt stress improved P_n_ and reduced T_r_, leading to the highest WUE_ins_ at leaf level. Moderate salt stress (SSC ≤ 0.6%) improved WUE_int_ at leaf level under both irrigation regimes. The PCA analysis showed that 2/3 of full irrigation without salt stress possessed the highest integrated WUE among all the treatments in both years; reduced irrigation coupled with mild salt stress (SSC = 0.2%) also achieved a relative higher comprehensive WUE in 2017. Soil salt content threshold for tomato caused by multiple salts was found to be below 0.2% and is lower than thresholds reported for nutrient solution.

## Author Contributions

HY carried out the experiments and finished the first manuscript. MS and TD supervised the work. XM and SK helped to edit the manuscript.

### Conflict of Interest Statement

The authors declare that the research was conducted in the absence of any commercial or financial relationships that could be construed as a potential conflict of interest.
